# New Construction of Maximum Distance Separable (MDS) Self-Dual Codes over Finite Fields

**DOI:** 10.3390/e21020101

**Published:** 2019-01-22

**Authors:** Aixian Zhang, Zhe Ji

**Affiliations:** Department of Mathematical Sciences, Xi’an University of Technology, Xi’an 710054, China

**Keywords:** MDS code, self-dual code, generalized reed-solomon code, extended generalized reed-solomon code

## Abstract

Maximum distance separable (MDS) self-dual codes have useful properties due to their optimality with respect to the Singleton bound and its self-duality. MDS self-dual codes are completely determined by the length n, so the problem of constructing *q*-ary MDS self-dual codes with various lengths is a very interesting topic. Recently X. Fang et al. using a method given in previous research, where several classes of new MDS self-dual codes were constructed through (extended) generalized Reed-Solomon codes, in this paper, based on the method given in we achieve several classes of MDS self-dual codes.

## 1. Introduction

Let $F_{q}$ be the finite field with *q* elements. A *q*-ary [n,k,d] linear code C is a *k*-dimensional subspace of $F_{q}^{n}$ with minimum (Hamming) distance d. If the parameters [n,k,d] satisfy k+d=n+1, the code is called an MDS (maximum distance separable) code. A self-dual code is a linear code satisfying $C=C^{⊥}.$ A linear complementary-dual code is a linear code satisfying $C\cap C^{⊥}=\left\{0\right\}.$

The study of MDS self-dual codes has attracted a great deal of attention in recent years due to its theoretical and practical importance. The center of the study of MDS codes includes the existence of MDS codes [1], classification of MDS codes [2], balanced MDS codes [3], non-Reed-Solomon MDS codes [4], complementary-dual MDS codes [5,6], and lowest density MDS codes [7].

As the parameters of an MDS self-dual code are completely determined by the code’s length *n*, the main interest here is to determine the existence and give the construction of *q*-ary MDS self-dual codes for various lengths. The problem is completely solved for the case where *q* is even [8]. Many MDS self-dual codes over finite fields of odd characteristics were constructed [9,10,11,12,13,14].

In [11], Jin and Xing constructed several classes of MDS self-dual code from generalized Reed-Solomon code. Yan generalized Jin and Xing’s method and constructed several classes of MDS self-dual codes via generalized Reed-Solomon codes and extended generalized Reed-Solomon codes [14]. In [12], Ladad, Liu and Luo produced more classes of MDS self-dual codes based on [11] and [14]. In [9], based on the [11,12,14] more new parameter MDS self-dual codes were presented. Based on the method raised in [9], we present some classes of MDS self-dual codes.

## 2. Preliminaries

In this section we introduce some basic notations of generalized Reed-Solomon codes and extended generalized Reed-Solomon codes. For more details, the reader is referred to [15].

Throughout this paper, *q* is a prime power, $F_{q}$ is the finite fields with *q* elements and let *n* be a positive integer with 1<n≤q. For any $x\in F_{q^{2}},$ we denote by $\bar{x}$ the conjugation of x. Given an [n,k,d] linear code C, its Euclidean dual code (resp. Hermitian dual code) is denoted by $C^{⊥}$ (resp. $C^{{⊥}_{H}}$). The codes $C^{⊥}$ and $C^{{⊥}_{H}}$ are defined by

$$C^{⊥}=\left\{x=\left(x_{1},x_{2},\ldots ,x_{n}\right)\in F_{q}^{n}:\sum_{i=1}^{n}x_{i}y_{i}=0,\forall y=\left(y_{1},y_{2},\ldots ,y_{n}\right)\in C\right\},$$

$$C^{{⊥}_{H}}=\left\{x=\left(x_{1},x_{2},\ldots ,x_{n}\right)\in F_{q^{2}}^{n}:\sum_{i=1}^{n}x_{i}\bar{y_{i}}=0,\forall y=\left(y_{1},y_{2},\ldots ,y_{n}\right)\in C\right\},$$

respectively. In this paper, we only consider the Euclidean inner product.

Let $\vec{a}=\left(\alpha_{1},\alpha_{2},\ldots ,\alpha_{n}\right),$ where $\alpha_{1},\alpha_{2},\ldots ,\alpha_{n}$ are *n* distinct elements of $F_{q}$. Fix *n* nonzero elements $v_{1},v_{2},\ldots ,v_{n}$ of $F_{q}$ ($v_{i}$ are not necessarily distinct), put $\vec{v}=\left(v_{1},v_{2},\ldots ,v_{n}\right).$ For 1≤k≤n, the *k*-dimensional generalized Reed-Solomon code (GRS for short) of length *n* associated with $\vec{a}$ and $\vec{v}$ is defined to be

(1) $${\mathrm{GRS}}_{k}\left(\vec{a},\vec{v}\right)=\left\{\left(v_{1}f\left(\alpha_{1}\right),v_{2}f\left(\alpha_{2}\right),\ldots ,v_{n}f\left(\alpha_{n}\right)\right):f\left(x\right)\in F_{q}\left[x\right],\deg\left(f\left(x\right)\right)\leq k-1\right\}.$$

It is well known that the code ${\mathrm{GRS}}_{k}\left(\vec{a},\vec{v}\right)$ is a *q*-ary [n,k,n−k+1] MDS code and the dual of a GRS code is again a GRS MDS code; indeed

$${\mathrm{GRS}}_{k}^{⊥}\left(\vec{a},\vec{v}\right)={\mathrm{GRS}}_{n-k}\left(\vec{a},{\vec{v}}^{\prime }\right)$$

for some ${\vec{v}}^{\prime }=\left(v_{1}^{\prime },v_{2}^{\prime },\ldots ,v_{n}^{\prime }\right)$ with $v_{i}^{\prime }\neq 0$ for all 1≤i≤n (e.g., see [15]).

Furthermore, the extended generalized Reed-Solomon code ${\mathrm{GRS}}_{k}\left(\vec{a},\vec{v},\infty \right)$ given by

(2) $${\mathrm{GRS}}_{k}\left(\vec{a},\vec{v},\infty \right)=\left\{\left(v_{1}f\left(\alpha_{1}\right),v_{2}f\left(\alpha_{2}\right),\ldots ,v_{n}f\left(\alpha_{n}\right),f_{k-1}\right):f\left(x\right)\in F_{q}\left[x\right],\deg\left(f\left(x\right)\right)\leq k-1\right\},$$

where $f_{k-1}$ stands for the coefficient of $x^{k-1}$ in f(x). It is also well known that ${\mathrm{GRS}}_{k}\left(\vec{a},\vec{v},\infty \right)$ is a *q*-ary [n+1,k,n−k+2] MDS code and the dual code is also a GRS MDS code (e.g., see [15]).

Put $\vec{a}=\left(\alpha_{1},\alpha_{2},\ldots ,\alpha_{n}\right)$ and denote by $A_{\vec{a}}$ the matrix

$$\begin{array}{c} \left(\begin{array}{cccc} 1 & 1 & \ldots & 1\\ \alpha_{1} & \alpha_{2} & \ldots & \alpha_{n}\\ \alpha_{1}^{2} & \alpha_{2}^{2} & \ldots & \alpha_{n}^{2}\\ \vdots & \vdots & \ddots & \vdots \\ \alpha_{1}^{n-2} & \alpha_{2}^{n-2} & \ldots & \alpha_{n}^{n-2} \end{array}\right) \end{array}$$

**Lemma** **1** ([11])**.** *The solution space of the equation system $A_{\vec{a}}X^{T}=0$ has dimension 1 and $\left\{\vec{u}=\left(u_{1},u_{2},\ldots ,u_{n}\right)\right\}$ is a basis of this solution space, where $u_{i}=\prod_{1\leq j\leq n,j\neq i}{\left(\alpha_{i}-\alpha_{j}\right)}^{-1}.$ Furthermore, for any two polynomials $f\left(x\right),g\left(x\right)\in F_{q}\left[x\right]$ with deg(f)≤k−1 and deg(g)≤n−k−1, one has $\sum_{i=1}^{n}f\left(\alpha_{i}\right)\left(u_{i}g\left(\alpha_{i}\right)\right)=0.$*

We define

$$L_{\vec{a}}\left(\alpha_{i}\right)=\prod_{1\leq j\leq n,j\neq i}\left(\alpha_{i}-\alpha_{j}\right).$$

The conclusion of the following lemma is straightforward. For completeness, we provide its proof.

**Lemma** **2** ([11])**.** Let n be an even number, if there exists $\lambda \in F_{q}^{*}$ such that $\lambda L_{\vec{a}}\left(\alpha_{i}\right)$ is square element for all i=1,2,…,n, then the code ${\mathrm{GRS}}_{n/2}\left(\vec{a},\vec{v}\right)$ defined in (1) is MDS self-dual code of length n.

**Proof.**  Let $f\left(x\right),g\left(x\right)\in F_{q}\left[x\right]$ with $\deg\left(f\right)\leq \frac{n}{2}-1$ and $\deg\left(g\right)\leq \frac{n}{2}-1.$ By Lemma 1, we have $\sum_{i=1}^{n}f\left(\alpha_{i}\right)\left(u_{i}g\left(\alpha_{i}\right)\right)=0,$ where $u_{i}=\prod_{1\leq j\leq n,j\neq i}{\left(\alpha_{i}-\alpha_{j}\right)}^{-1}$ for i=1,2,…,n. Hence,

$$0=\lambda \sum_{i=1}^{n}f\left(\alpha_{i}\right)\left(u_{i}g\left(\alpha_{i}\right)\right)=\sum_{i=1}^{n}f\left(\alpha_{i}\right)\left(\lambda u_{i}g\left(\alpha_{i}\right)\right)=\sum_{i=1}^{n}\left(v_{i}f\left(\alpha_{i}\right)\right)\;\;\;\left(v_{i}g\left(\alpha_{i}\right)\right)\left(\mathrm{since}\;\lambda u_{i}=v_{i}^{2}\right).$$

This implies that ${\mathrm{GRS}}_{n/2}^{⊥}\left(\vec{a},\vec{v}\right)={\mathrm{GRS}}_{n/2}\left(\vec{a},\vec{v}\right).$ □

H. Yan [14] observed the following two results.

**Lemma** **3** ([14])**.** *Let n be an even integer and $k=\frac{n}{2}.$ If $-L_{\vec{a}}\left(\alpha_{i}\right)$ is square element for all i=1,2,…,n−1, then the code ${\mathrm{GRS}}_{k}\left(\vec{a},\vec{v},\infty \right)$ defined in (2) is MDS self-dual code of length n.*

**Lemma** **4** ([14])**.** *Let m∣q−1 be a positive integer and let $\alpha \in F_{q}$ be a primitive m-th root of unity. Then for any 1≤i≤m, we have*

$$\prod_{1\leq j\leq m,j\neq i}\left(\alpha^{i}-\alpha^{j}\right)=m\alpha^{-i}.$$

## 3. Main Result

Let $q=r^{2},$ where *r* is odd prime power, $F_{q}$ be the finite fields with *q* elements. Suppose m∣q−1,α is a primitive *m*-th root of unity and H=<β> is the cyclic group generated by β.

**Theorem** **1.** *Let $q=r^{2},$ where r is an odd prime power, r≡1(mod4). Suppose that m∣(q−1) and $\frac{q-1}{m}$ is even, m≡0(mod4). If $1\leq t\leq \frac{2(r+1)}{gcd(2(r+1),m)}.$ Then there exists an $\left[n=tm,\frac{n}{2}\right]$-MDS self-dual code.*

**Proof.**  Let α be a primitive *m*-th root of unity and H=<β> is the cyclic group of order 2(r+1). By the theorem of group homomorphism,

(H×〈α〉)/〈α〉≅H/(H∩〈α〉).

Let $i_{1},i_{2},\ldots ,i_{t}$ be *t* distinct elements, such that $0\leq i_{1}<i_{2}<\cdots <i_{t}<2\left(r+1\right).$ Denote $I=\left\{i_{1},i_{2},\ldots ,i_{t}\right\},A=i_{1}+i_{2}+\cdots +i_{t}$ and $B=\{\beta^{i_{1}},\beta^{i_{2}},\ldots ,\beta^{i_{t}}\}$ be a set of coset representatives of (H×〈α〉)/〈α〉. Let

$$\vec{a}=\left(\alpha \beta^{i_{1}},\ldots ,\alpha^{m}\beta^{i_{1}},\alpha \beta^{i_{2}},\ldots ,\alpha^{m}\beta^{i_{2}},\ldots ,\alpha \beta^{i_{t}},\ldots ,\alpha^{m}\beta^{i_{t}}\right).$$

Then the entries of $\vec{a}$ are distinct in $F_{q}^{*}.$

It is known that $x^{m}-y^{m}=\prod_{j=1}^{m}\left(x-\alpha^{j}y\right)$. By the statement of Lemma 3, we get

$$\begin{array}{lll} L_{\vec{a}}\left(\beta^{z}\alpha^{k}\right) & = & \prod_{1\leq j\leq m,j\neq k}\left(\beta^{z}\alpha^{k}-\beta^{z}\alpha^{j}\right)\prod_{l\in I,l\neq z}\prod_{j=1}^{m}\left(\beta^{z}\alpha^{k}-\beta^{l}\alpha^{j}\right)\\ & = & \beta^{z(m-1)}\prod_{1\leq j\leq m,j\neq k}\left(\alpha^{k}-\alpha^{j}\right)\prod_{l\in I,l\neq z}\left[{\left(\beta^{z}\alpha^{k}\right)}^{m}-\beta^{lm}\right]\\ & = & \beta^{z(m-1)}m\alpha^{-k}\prod_{l\in I,l\neq z}\left(\beta^{zm}-\beta^{lm}\right). \end{array}$$

Let $v=\prod_{l\in I,l\neq z}\left(\beta^{zm}-\beta^{lm}\right),$ then

$$\begin{array}{lll} v^{r} & = & \prod_{l\in I,l\neq z}\left(\beta^{zmr}-\beta^{lmr}\right)\;\;\;\left(\mathrm{since}\;\beta^{2(r+1)}=1,\beta^{r}=-\beta^{-1}\right)\\ & = & \prod_{l\in I,l\neq z}\left[{\left(-\beta^{-1}\right)}^{zm}-{\left(-\beta^{-1}\right)}^{lm}\right]\\ & = & \prod_{l\in I,l\neq z}\left[{\left(\beta^{-1}\right)}^{zm}-{\left(\beta^{-1}\right)}^{lm}\right]\\ & = & \prod_{l\in I,l\neq z}{\left(\beta^{-1}\right)}^{zm+lm}\left(\beta^{lm}-\beta^{zm}\right)\\ & = & {\left(-1\right)}^{t-1}\beta^{-(A+(t-2)z)m}v \end{array}$$

So $v^{r-1}={\left(-1\right)}^{t-1}\beta^{-(A+(t-2)z)m}.$

Let *g* be a generator of $F_{q}^{*}$, then $\alpha =g^{\frac{q-1}{m}},\beta =g^{\frac{r-1}{2}},-1=g^{\frac{r^{2}-1}{2}},v=g^{\frac{r+1}{2}\left(t-1\right)-\left(A+\left(t-2\right)z\right)\frac{m}{2}+i\left(r+1\right)}.$ Note that β,m and α are square elements of $F_{q}^{*},$ we take $\lambda =g^{\frac{r+1}{2}\left(t-1\right)},$ then $\lambda L_{\vec{a}}\left(\beta^{z}\alpha^{k}\right)$ is a square element of $F_{q}^{*}$.

This implies there exists a *q*-ary $\left[n,\frac{n}{2}\right]$ MDS self-dual code. □

**Example** **1.** *Let $r=173,q=173^{2},r\equiv 1\left(\mathrm{mod}4\right),m=4\times 43,\frac{q-1}{m}=174$ is even. For $1\leq t\leq \frac{2(r+1)}{gcd(2(r+1),m)}=87,$ we choose t=81. By Theorem 1, there exists the MDS self-dual code with length n=mt=* 13,932.

**Theorem** **2.** *Let $q=r^{2},$ where r is an odd prime power. Suppose that m is odd, m∣(q−1) and $\frac{q-1}{m}$ is even. If $1\leq t\leq min\{\frac{r+1}{gcd(2(r+1),m)},\frac{r+1}{2}\}$ and t is odd, then there exists a q-ary $\left[n=tm+1,\frac{n}{2}\right]$ MDS self-dual code over $F_{q}.$*

**Proof.**  Let α and β be the same as in Theorem 1, we choose *t* distinct even number $i_{1},i_{2},\ldots ,i_{t}$, $0\leq i_{1}<i_{2}<\cdots <i_{t}<2\left(r+1\right).$ Denote $I=\left\{i_{1},i_{2},\cdots ,i_{t}\right\},A=i_{1}+i_{2}+\ldots +i_{t}$. Suppose all $i_{j}\equiv 2\left(\mathrm{mod}4\right),j=1,2,\cdots ,t.$ The proof is as similar as in Theorem 1. We get

$$L_{\vec{a}}\left(\beta^{z}\alpha^{k}\right)=\beta^{z(m-1)}m\alpha^{-k}\prod_{l\in I,l\neq z}\left(\beta^{zm}-\beta^{lm}\right).$$

Let $v=\prod_{l\in I,l\neq z}\left(\beta^{zm}-\beta^{lm}\right),$ then we get

$$v^{r-1}={\left(-1\right)}^{t-1}\beta^{-(A+(t-2)z)m},v=g^{\frac{r+1}{2}\left(t-1\right)-\frac{(A+(t-2)z)m}{2}+i\left(r+1\right)},$$

since $\frac{A+(t-2)z}{2}$ is even, it implies that *v* is a square element of $F_{q}^{*}.$ So $-L_{\vec{a}}\left(\beta^{z}\alpha^{k}\right)$ is square element of $F_{q}^{*}$. By Lemma 3, there exists a *q*-ary $\left[n,\frac{n}{2}\right]$ MDS self-dual code. □

**Example** **2.** *Let $r=67,q=67^{2},m=11,\frac{q-1}{m}=408$ is even. Since 2(r+1)=136=4×34, for $1\leq t\leq \frac{r+1}{gcd(2(r+1),m)}=68,$ we choose t=27. By Theorem 2, there exists the MDS self-dual code with length n=mt+1=298.*

**Theorem** **3.** *Let $q=r^{2},$ where r is an odd prime power, r≡1(mod4). Suppose that m is odd, m∣(q−1) and $\frac{q-1}{m}$ is even. If $1\leq t\leq min\{\frac{r+1}{gcd(2(r+1),m)},\frac{r+1}{2}\}$ and t is odd, then there exists a q-ary $\left[n=tm+1,\frac{n}{2}\right]$ MDS self-dual code over $F_{q}$.*

**Proof.**  Let α and β be the same as in Theorem 1, we choose *t* distinct even number $i_{1},i_{2},\ldots ,i_{t}$, $0\leq i_{1}<i_{2}<\cdots <i_{t}<2\left(r+1\right).$ Denote $I=\left\{i_{1},i_{2},\cdots ,i_{t}\right\},A=i_{1}+i_{2}+\ldots +i_{t}$, and $i_{j}\equiv 2\left(\mathrm{mod}4\right),j=1,2,\cdots ,t.$ We define the generalized Reed -Solomon code ${\mathrm{GRS}}_{k}\left(\vec{a},\vec{v}\right)$ with

$$\vec{a}=\left(0,\alpha \beta^{i_{1}},\ldots ,\alpha^{m}\beta^{i_{1}},\alpha \beta^{i_{2}},\ldots ,\alpha^{m}\beta^{i_{2}},\ldots ,\alpha \beta^{i_{t}},\ldots ,\alpha^{m}\beta^{i_{t}}\right).$$

For any z∈I and 1≤k≤m, we get

$$\begin{array}{ccc} L_{\vec{a}}\left(\beta^{z}\alpha^{k}\right) & = & \beta^{z}\alpha^{k}\prod_{1\leq j\leq m,j\neq k}\left(\beta^{z}\alpha^{k}-\beta^{z}\alpha^{j}\right)\prod_{l\in I,l\neq z}\prod_{j=1}^{m}\left(\beta^{z}\alpha^{k}-\beta^{l}\alpha^{j}\right)\\ & = & \beta^{zm}m\prod_{l\in I,l\neq z}\left(\beta^{zm}-\beta^{lm}\right) \end{array}$$

and

$$L_{\vec{a}}\left(0\right)=\prod_{l\in I}\prod_{j=1}^{m}\left(0-\beta^{l}\alpha^{j}\right)={\left(-1\right)}^{mt}\alpha^{\frac{m(m+1)}{2}}{\left(\prod_{l\in I}\beta^{l}\right)}^{m}.$$

Since $r\equiv 1\left(\mathrm{mod}4\right),\frac{q-1}{m}$ is even, so α,β,m,−1 are square elements of $F_{q}^{*},$ we only need to consider $v=\prod_{l\in I,l\neq z}\left(\beta^{zm}-\beta^{lm}\right).$ As the calculation in the proof of Theorem 1, $v=g^{\frac{r+1}{2}\left(t-1\right)-\frac{(A+(t-2)z)m}{2}+i\left(r+1\right)}.$ Since all $i_{j}\equiv 2\left(\mathrm{mod}4\right)$ and *t* is odd, so $\frac{(A+(t-2)z)m}{2}$ is even. $L_{\vec{a}}\left(\beta^{z}\alpha^{k}\right)$, $L_{\vec{a}}\left(0\right)$ are square elements of $F_{q}^{*}.$ By Lemma 2, there exists a *q*-ary $\left[n,\frac{n}{2}\right]$ MDS self-dual code. □

**Example** **3.** *Let $r=101,r\equiv 1\left(\mathrm{mod}4\right),q=101^{2},m=75,\frac{q-1}{m}=136$ is even. Since 2(r+1)=204=4×51, for $1\leq t\leq \frac{r+1}{gcd(2(r+1),m)}=34,$ we choose t=33. By Theorem 2, there exists the MDS self-dual code with length n=mt+1=2476.*

**Theorem** **4.** *Let $q=r^{2},$ where r is an odd prime power. Suppose that $m|\left(q-1\right),\frac{q-1}{m}$ is even. If $1\leq t\leq \frac{2(r+1)}{gcd(2(r+1),m)}$ and tm is even, then there exists a q-ary $\left[n=tm+2,\frac{n}{2}\right]$ MDS self-dual code over $F_{q}$.*

**Proof.**  Let α and β be the same as in Theorem 1. We define the extended generalized Reed -Solomon code ${\mathrm{GRS}}_{k}\left(\vec{a},\vec{v},\infty \right)$ with

$$\vec{a}=\left(0,\alpha \beta^{i_{1}},\cdots ,\alpha^{m}\beta^{i_{1}},\alpha \beta^{i_{2}},\cdots ,\alpha^{m}\beta^{i_{2}},\cdots ,\alpha \beta^{i_{t}},\cdots ,\alpha^{m}\beta^{i_{t}}\right).$$

For any z∈I and 1≤k≤m, we get

$$\begin{array}{ccc} L_{\vec{a}}\left(\beta^{z}\alpha^{k}\right) & = & \beta^{z}\alpha^{k}\prod_{1\leq j\leq m,j\neq k}\left(\beta^{z}\alpha^{k}-\beta^{z}\alpha^{j}\right)\prod_{l\in I,l\neq z}\prod_{j=1}^{m}\left(\beta^{z}\alpha^{k}-\beta^{l}\alpha^{j}\right)\\ & = & \beta^{zm}m\prod_{l\in I,l\neq z}\left(\beta^{zm}-\beta^{lm}\right) \end{array}$$

and

$$L_{\vec{a}}\left(0\right)=\prod_{l\in I}\prod_{j=1}^{m}\left(0-\beta^{l}\alpha^{j}\right)={\left(-1\right)}^{mt}\alpha^{\frac{m(m+1)}{2}}{\left(\prod_{l\in I}\beta^{l}\right)}^{m}.$$

**Case 1**: If *m* is even, *t* is odd.

$\beta^{zm},m$ and $L_{\vec{a}}\left(0\right)$ are square elements of $F_{q}^{*}.$ Let $v=\prod_{l\in I,l\neq z}\left(\beta^{zm}-\beta^{lm}\right),$ as the calculation in Theorem 1, $v=g^{\frac{r+1}{2}\left(t-1\right)-\frac{(A+(t-2)z)m}{2}+i\left(r+1\right)}.$ So we only need to consider the parity of $\frac{(A+(t-2)z)m}{2}.$

- $i_{1},i_{2},\ldots ,i_{t}$ are even number, so A+(t−2)z≡0(mod2),v is a square element of $F_{q}^{*}.$
- $i_{1},i_{2},\ldots ,i_{t}$ are odd number, so A+(t−2)z≡0(mod2),v is a square element of $F_{q}^{*}.$

**Case 2**: If *m* and *t* are even, r≡3(mod4), we assume *A* is an even integer. It follows that $\frac{r+1}{2}\left(t-1\right)-\frac{(A+(t-2)z)m}{2}$ is an even integer.

**Case 3**: If *m* is odd, *t* is even.

- t≡0(mod4)
  (1) If r≡1(mod4), all $i_{1},i_{2},\ldots ,i_{t}$ are odd, and A≡0(mod4), then then (r+1)(t−1)−(A+(t−2)z)m≡0(mod4),v is a square element of $F_{q}^{*}.$
  (2) If r≡3(mod4), all $i_{1},i_{2},\ldots ,i_{t}$ are even, and A≡2(mod4), then (r+1)(t−1)−(A+(t−2)z)m≡0(mod4),v is a square element of $F_{q}^{*}.$
- t≡2(mod4).
  (1) If r≡1(mod4),A≡2(mod4), then (r+1)(t−1)−(A+(t−2)z)m≡0(mod4),v is square of $F_{q}^{*}.$
  (2) If r≡3(mod4),A≡0(mod4), then (r+1)(t−1)−(A+(t−2)z)m≡0(mod4),v is square of $F_{q}^{*}.$

 □

We can extend the Theorem 1 to a more general case.

**Theorem** **5.** *Let $q=r^{2},$ where r is an odd prime power. Suppose that $m|\left(q-1\right),\frac{q-1}{m}$ is even, s∣m,s∣r−1 and $\frac{r-1}{s}$ is even. If $1\leq t\leq \frac{s(r+1)}{gcd(s(r+1),m)},$ then there exists a q-ary $\left[n=tm,\frac{n}{2}\right]$ MDS self-dual code over $F_{q}.$*

**Proof.**  Let α be a primitive *m*-th root of unity and H=<β> is the cyclic group of order s(r+1). By the theorem of group homomorphism,

(H×〈α〉)/〈α〉≅H/(H∩〈α〉),

Let $i_{1},i_{2},\ldots ,i_{t}$ be *t* distinct elements, such that $0\leq i_{1}<i_{2}<\cdots <i_{t}<2\left(r+1\right).$ Denote $I=\left\{i_{1},i_{2},\ldots ,i_{t}\right\},A=i_{1}+i_{2}+\ldots +i_{t}$ and $B=\{\beta^{i_{1}},\beta^{i_{2}},\ldots ,\beta^{i_{t}}\}$ be a set of coset representatives of H×〈α〉. Let

$$\vec{a}=\left(\alpha \beta^{i_{1}},\cdots ,\alpha^{m}\beta^{i_{1}},\alpha \beta^{i_{2}},\cdots ,\alpha^{m}\beta^{i_{2}},\cdots ,\alpha \beta^{i_{t}},\cdots ,\alpha^{m}\beta^{i_{t}}\right).$$

Similar with Theorem 1, we get

$$\begin{array}{ccc} L_{\vec{a}}\left(\beta^{z}\alpha^{k}\right) & = & \prod_{1\leq j\leq m,j\neq k}\left(\beta^{z}\alpha^{k}-\beta^{z}\alpha^{j}\right)\prod_{l\in I,l\neq z}\prod_{j=1}^{m}\left(\beta^{z}\alpha^{k}-\beta^{l}\alpha^{j}\right)\\ & = & \beta^{z(m-1)}\cdot m\cdot \alpha^{-k}\prod_{l\in I,l\neq z}\left(\beta^{zm}-\beta^{lm}.\right) \end{array}$$

Since $\beta^{s(r+1)}=1,$ then $\beta^{r+1}=\xi_{s},$ where $\xi_{s}$ is *s*-th primitive root of unity. So $\beta^{r}=\xi_{s}\beta^{-1}.$ Let $v=\prod_{l\in I,l\neq z}\left(\beta^{zm}-\beta^{lm}\right).$ Since s∣m, then

$$\begin{array}{ccc} v^{r} & = & \prod_{l\in I,l\neq z}\left({\left(\beta^{-1}\right)}^{zm}-{\left(\beta^{-1}\right)}^{lm}\right)\\ & = & \prod_{l\in I,l\neq z}\beta^{-(l+z)m}\left(\beta^{lm}-\beta^{zm}\right)\\ & = & {\left(-1\right)}^{t-1}\beta^{-(A+(t-2)z)m}v. \end{array}$$

So $v^{r-1}={\left(-1\right)}^{t-1}\beta^{-(A+(t-2)z)m}.$

Let *g* be a generator of $F_{q}^{*}.$ It follows that $\beta =g^{\frac{r-1}{s}}$ and $-1=g^{\frac{r^{2}-1}{2}}.$ So

$$v=g^{\frac{\left(r+1\right)}{2}\left(t-1\right)-\left[A+\left(t-2\right)z\right]\frac{m}{s}}.$$

**Case 1**: If *m* odd and *t* even, we can take $\lambda =g^{\frac{\left(r+1\right)}{2}\left(t-1\right)-A\cdot \frac{m}{s}}.$ Hence, we have $\lambda L_{\vec{a}}\left(\beta^{z}\alpha^{k}\right)$ is square element of $F_{q}^{*}.$

**Case 2**: If *m* even and $2|\frac{m}{s}$, we can take $\lambda =g^{\frac{\left(r+1\right)}{2}\left(t-1\right)}.$ Hence, we have $\lambda L_{\vec{a}}\left(\beta^{z}\alpha^{k}\right)$ is square element of $F_{q}^{*}.$

So there exists a *q*-ary MDS self-dual code with length n.  □

## 4. Conclusions

In this paper, based on the method from [9], we construct several classes of MDS self-dual code over finite fields with odd characteristics via the generalized Reed-Solomon code and extend the generalized Reed-Solomon code.
